# CNOT2 promotes degradation of p62/SQSTM1 as a negative regulator in ATG5 dependent autophagy

**DOI:** 10.18632/oncotarget.17682

**Published:** 2017-05-08

**Authors:** Kwon Jeong, Hee Young Kwon, Myoung Seok Jeong, Eun Jung Sohn, Sung-Hoon Kim

**Affiliations:** ^1^ Cancer Molecular Targeted Herbal Research Center, College of Korean Medicine, Kyung Hee University, 1 Hoegi-Dong, Dongdaemun-Gu, Seoul 130-701, Republic of Korea

**Keywords:** CNOT2, p62/SQSTM1, ATG5, PB1 domain, autophagy

## Abstract

Though CNOT2 is involved in regulation of adipogenic differentiation, apoptotic cell death and metastasis, the underlying autophagic mechanism of CNOT2 was unknown until now. Thus, in the present study, the critical role of CNOT2 in autophagy was elucidated in association with p62/SQSTM1 signaling. CNOT2 depletion induced p62/SQSTM1 accumulation and LC3B-II conversion, and also increased the number of puncta with impaired autophagic flux. In contrast, CNOT2 overexpression induced downregulation and ubiquitination of p62/SQSTM1 in HEK293 QBI. Furthermore, ubiquitination of p62/SQSTM1 was blocked by autophagy inhibition. Interestingly, CNOT2 was correlated with p62/SQSTM1 in HEK293 QBI cells and also was colocalized with p62/SQSTM1 in H1299 cells. Additionally, ATG5 was upregulated in CNOT2-depleted H1299 cells, while degradation of p62/SQSTM1 by CNOT2 was detected in *ATG5^+/+^* MEF cells but not in *ATG5^−/−^* MEF cells. Of note, CNOT2 induced degradation of p62/SQSTM1 in HEK293 QBI cells co-transfected with Myc-ΔLIR/KIR or Myc-ΔUBA, but not with Myc-ΔPB1. Sub G_1_ population was increased in CNOT2-depleted H1299 cells by late autophagy inhibitors, ammonium chloride and chloroquine compared to 3-methyladenine. Overall, these findings provide novel insight into the critical role of CNOT2 as a negative regulator in ATG5 dependent autophagy.

## INTRODUCTION

Autophagy is a catabolic process, where misfolded proteins or damaged organelles are recycled to maintain cellular homeostasis mainly by various factors such as nutrient deprivation, infection, aging and others [1–3]. Generally, the processes of autophagy machinery are the formation of isolation membrane, its elongation by engulfing some parts of the cytoplasm, and the formation of autophagosome as a double-membrane vesicle [4]. Emerging evidences reveal that the outer membrane of the autophagosome fuses with the lysosome to form the autophagolysosome to remove the luminal materials for survival [3–5].

Among 30 autophagy related genes (ATGs), their four subgroups play pivotal roles in autophagy processes; the ULK complex, the class III phosphatidylinositol 3-kinase (PI3K)/VPS34 complex I, two ubiquitin-like protein (ATG12 and ATG8/LC3) conjugation systems, and ATG9-ATG18 complexes [6].

Especially, p62/SQSTM1, one of autophagy-specific substrates, is degraded through the autophagy-lysosomal pathway [7], while p62/SQSTM1 accumulation often enhances tumor growth and other pathological conditions [2, 8–10]. It is well documented that p62/SQSTM1 is closely interacted with variety of molecules such as PKC delta, RIP, Raptor, TRAF6, Keap1, NFkB, Nrf2, ATG8/LC3 and poly ubiquitinated proteins [11–17]. Nevertheless, the negative regulators of p62/SQSTM1 accumulation still remain unclear so far.

The carbon catabolite repressed 4 (CCR4) negative on TATA-less (NOT) complex (>2 MDa) acts to regulate gene expression involved in cell cycle control, degradation of mRNAs in P-bodies [18–21]. CCR4-NOT complex (CNOT) consists of eleven subunits as a master regulator of mRNA stability, transcription, translation and mRNA export [20, 22, 23]. Among them, human CNOT2 is known to regulate the deadenylase activity and structural integrity of the CCR4–NOT complex and control embryonic development in *C. elegans* and *D. melanogaster* [20, 21, 24]. Furthermore, CNOT2 is critically involved in regulation of apoptotic cell death [18], metastasis [25] and adipogenic differentiation [26].

Nevertheless, the underlying autophagic mechanism of CNOT2 was not reported until now. Thus, in the current study, the role of CNOT2 was investigated in association with p62/SQSTM1-degradation as an autophagy regulator.

## RESULTS

### Depletion of CNOT2 inhibits autophagic flux

**Figure 1:**
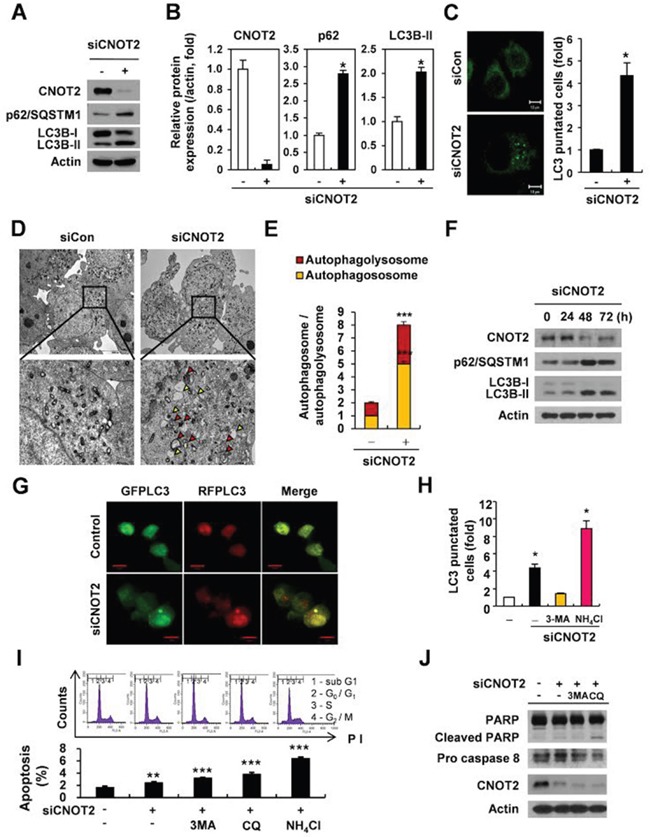
Depletion of CNOT2 induces autophagy via accumulation of p62/SQSTM1 and LC3B-II conversion, LC3 fluorescent puncta and autophagosomes, but impairs autophagic flux in H1299 cells **(A)** Western blotting was conducted for p62/SQSTM1 and LC3B-II in H1299 cells transfected with CNOT2 siRNA. **(B)** ImageJ densitometric analysis of the relative CNOT2, p62/SQSTM1 and LC3B-II protein expression levels (means ± SD of 3 independent experiments, *p < 0.05 vs untreated control by Student *t* test). **(C)** Accumulation of LC3 fluorescent puncta in H1299 cells transfected with CNOT2 siRNA compared to untreated control in H1299 cells (means ± SD of 3 independent experiments, * *p* < 0.05 vs untreated control by Student *t* test). **(D)** A number of autophagosomes and autophagolysosomes were observed in H1299 cells transfected with CNOT2 siRNA by TEM. The below panel is the enlarged image for the black frame. Autophagosomes and autophagolysosomes were represented by white and black arrowheads, respectively. Scale bar regular image: 10 μm, enlarge image: 2 μm. **(E)** Quantitative analysis of autohagosome and autophagolysosome in H1299 cells transfected with CNOT2 siRNA (means ± SD of 3 independent experiments, *** *p* < 0.001 vs untreated control by Student *t* test). **(F)** Effect of CNOT2 depletion on the expression of p62 and LC3B-II in CNOT2 depleted H1299 cells in a time course. **(G)** Depletion of CNOT2 inhibited autophagic flux in H1299 cells. Representative confocal images were exhibited in H1299 cells co-transfected with siCNOT2 and RFP-LC3 and GFP-LC3 constructs. Autophagosome was visualized as yellow or orange puncta (RFP-GFP-LC3B) in merged images, whereas red puncta (RFP-LC3B) represents autophagolysosome, since acidification reduces green fluorescence. Scale bar: 10 μm. **(H)** Quantitative analysis of LC3-puncta in H1299 cells transfected with CNOT2 siRNA in the present or absence of autophagy inhibitor 3-MA or NH_4_Cl (means ± SD of 3 independent experiments, * *p* < 0.05 vs untreated control by Student *t* test). **(I)** Effect of autophagy inhibitors on sub G_1_ population in CNOT2-depleted H1299 cells. Cells were transfected with CNOT2 siRNA for 24 h and treated with 3-MA, CQ and NH_4_Cl for another 24 h. Then the cells were fixed with 75 % ethanol and stained with 50 mg/ml of PI. The cell cycle analysis was performed using FACs Calibur, and the data were analyzed using CellQuest Software (means ± SD of 3 independent experiments, ** *p* < 0.01, *** *p* < 0.001 vs untreated control by Student *t* test). **(J)** Effect of autophagy inhibitors on PAPR cleavage and procaspase 8 in H1299 cells transfected with CNOT2 siRNA. Cells were transfected with CNOT2 siRNA for 48 h, exposed to 3MA or CQ for 24 h and Western blotting was conducted with antibodies of PAPR cleavage, procaspase 8, CNOT2 and actin.

Autophagy is a catabolic process by which the cells break down their polyubiquitinated protein aggregates that are not yet degraded through the proteasomal pathway [27–29]. In particular, the autophagy adaptor protein p62/SQSTM1 recognizes polyubiquitinated protein aggregates and incorporates them into autophagosomes via direct interaction with LC3B-II on the autophagosomal membrane, thereby delivering the aggregates for degradation [30]. In the present study, depletion of CNOT2 induced p62/SQSTM1 accumulation and LC3B-II conversion, as biochemical markers of autophagy, in H1299 cells (Figure 1A and 1B). As shown in Figure 1C, the puncta pattern of LC3B-II fluorescence was detected in CNOT2-depleted H1299 cells, while a diffuse localization of LC3B-II fluorescence was observed in control group cells. Consistently, autophagic vacuoles, autophagosome (yellow arrowheads) and autophagolysosomes (red arrowheads) were observed by electron microscopy in CNOT2 siRNA transfected H1299 cells (Figure 1D and 1E). Also, turnover assay revealed that p62/SQSTM1 was accumulated at 48 h and then tended to be degraded from 72 h in CNOT2 depleted H1299 cells (Figure 1F). Next, it was examined whether or not CNOT2 completely induces autophagic flux in CNOT2-depleted H1299 cells. As shown in Figure 1G, depletion of CNOT2 inhibited the autophagic flux with yellow color, when autophagosomal green puncta and autophagolysosomal red puncta were merged in CNOT2-depleted H1299 cells. Furthermore, it was investigated how CNOT2 regulates autophagic flux by using autophagy inhibitors. We blocked lysosomal degradation by using ammonium chloride (NH_4_Cl) as previously reported [31, 32]. The formation of puncta in CNOT2-depleted H1299 cells was inhibited in the presence of early stage autophagy inhibitor 3-MA compared to untreated control, whereas the number of puncta was increased in late stage autophagy inhibitor NH_4_Cl treated H1299 cells (Figure 1H). To clarify the effect of autophagy inhibitors such as 3-MA, CQ and NH_4_Cl on the fate of H1299 cells, FACS cell cycle analysis was performed. As shown in Figure 1I, cell cycle analysis revealed that increased sub G_1_ population was detected in CNOT2 depleted H1299 cells by 3-MA, CQ and NH_4_Cl treatment in order. And PARP was cleaved and procaspase 8 was attenuated by CQ better than 3-MA (Figure 1J).

### CNOT2 induces ubiquitination and degradation of p62/SQSTM1 in HEK293 QBI cells

**Figure 2:**
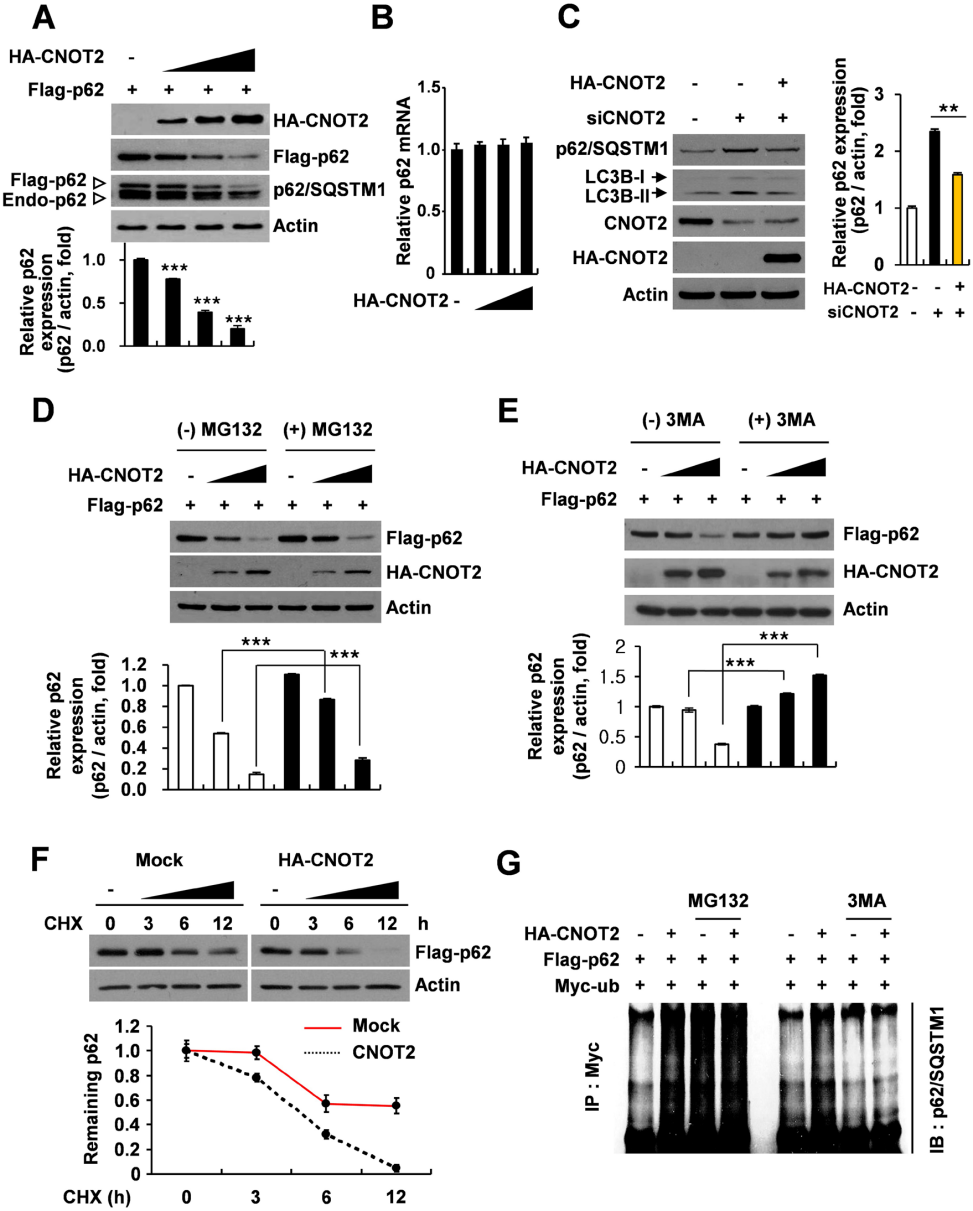
CNOT2 increases ubiquitination and degradation of p62/SQSTM1 protein in HEK293 QBI cells HEK293 QBI cells were co-transfected with Flag-p62 and HA-CNOT2 plasmids. Thereafter, the cells were cultured for 48 h and subjected to **(A)** western blot or **(B)** RT-PCR (means ± SD of 3 independent experiments, *** *p* < 0.001 vs control by Student *t* test). **(C)** Cells were transiently transfected with CNOT2 siRNA for 48 h and then subjected to immunoblotting with indicated antibodies (means ± SD of 3 independent experiments, ** *p* < 0.01 vs control by Student *t* test). **(D)** HEK293 QBI cells were transiently transfected with Flag-p62 and HA-CNOT2 plasmids and incubated for 48 h in the presence or absence of MG132 (means ± SD of 3 independent experiments, *** *p* < 0.001 vs untreated control by Student *t* test). **(E)** HEK293 QBI cells were transiently transfected with Flag-p62 and HA-CNOT2 plasmids and incubated for 48 h in the presence or absence of 3-MA. Cell lysates were immunoblotted with indicated antibodies (means ± SD of 3 independent experiments, ** *p* < 0.01; *** *p* < 0.001 vs untreated control by Student *t* test). **(F)** HEK293 QBI cells were transiently transfected with HA-CNOT2 plasmid or empty vector in the presence or absence of CHX. **(G)** HEK293 QBI cells were transiently co-transfected with HA-CNOT2, Flag-p62, and Myc-ubiquitin plasmids in the presence or absence of MG132 and 3-MA. Ubiquitination of p62 was evaluated with anti-p62/SQSTM1 antibody.

Given that CNOT2, as one of subunits of CCR4-NOT complex, modulates mRNA degradation and transcriptional regulation [22, 23, 33], the underlying functional protein-protein interactions between CNOT2 and p62/SQSTM1 were explored in HEK293 QBI cells. First, the effect of CNOT2 using HA-CNOT2 plasmid was evaluated on p62/SQSTM1 in HEK293 QBI cells. As shown in Figure 2A, the expression level of p62/SQSTM1 was decreased by CNOT2 overexpression in a dose-dependent manner, while CNOT2 did not affect p62/SQSTM1 mRNA level (Figure 2B). Conversely, depletion of CNOT2 induced p62/SQSTM1 accumulation and LC3B-II conversion, which was suppressed by CNOT2 overexpression (Figure 2C). To examine whether p62/SQSTM1 was downregulated by CNOT2 overexpression, HEK293 QBI cells were co-transfected with HA-CNOT2 and Flag-p62 plasmids and then exposed to MG132 (proteasome inhibitor) or 3-MA (autophagy inhibitor). Immunoblotting revealed that downregulation of p62/SQSTM1 by CNOT2 was slightly reduced by MG132 (Figure 2D), whereas p62/SQSTM1 was rather accumulated by 3-MA (Figure 2E), indicating that downregulation of p62/SQSTM1 by CNOT2 is mediated via autophagic degradation. To further ascertain the regulatory role of the degradation pathways, the steady levels of p62/SQSTM1 by protein synthesis inhibitor cycloheximide (CHX) treatment were evaluated in the intact or overexpressed CNOT2 cells. The expression of HA-CNOT2 accelerated Flag-p62 expression decay, whereas empty vector stabilized flag-p62. As shown in Figure 2F, downregulation of p62/SQSTM1 by CNOT2 was enhanced by CHX treatment in a time-dependent manner in HEK293 QBI cells. To confirm whether downregulation of p62/SQSTM1 is mediated by ubiquitination, ubiquitination assay was conducted in HEK293 QBI cells co-transfected with HA-CNOT2, Flag-p62 and Myc-ubiquitin (Myc-ub) plasmids in the presence or absence of 3-MA or MG132. As shown in Figure 2G, CNOT2 overexpression induced ubiquitination of p62/SQSTM1, which was blocked by early stage autophagy inhibitor 3-MA, but not MG132, indicating autophagic degradation of p62/SQSTM1 by CNOT2.

### CNOT2 binds to p62/SQSTM1 in HEK293 QBI cells as a key regulator of autophagy

**Figure 3:**
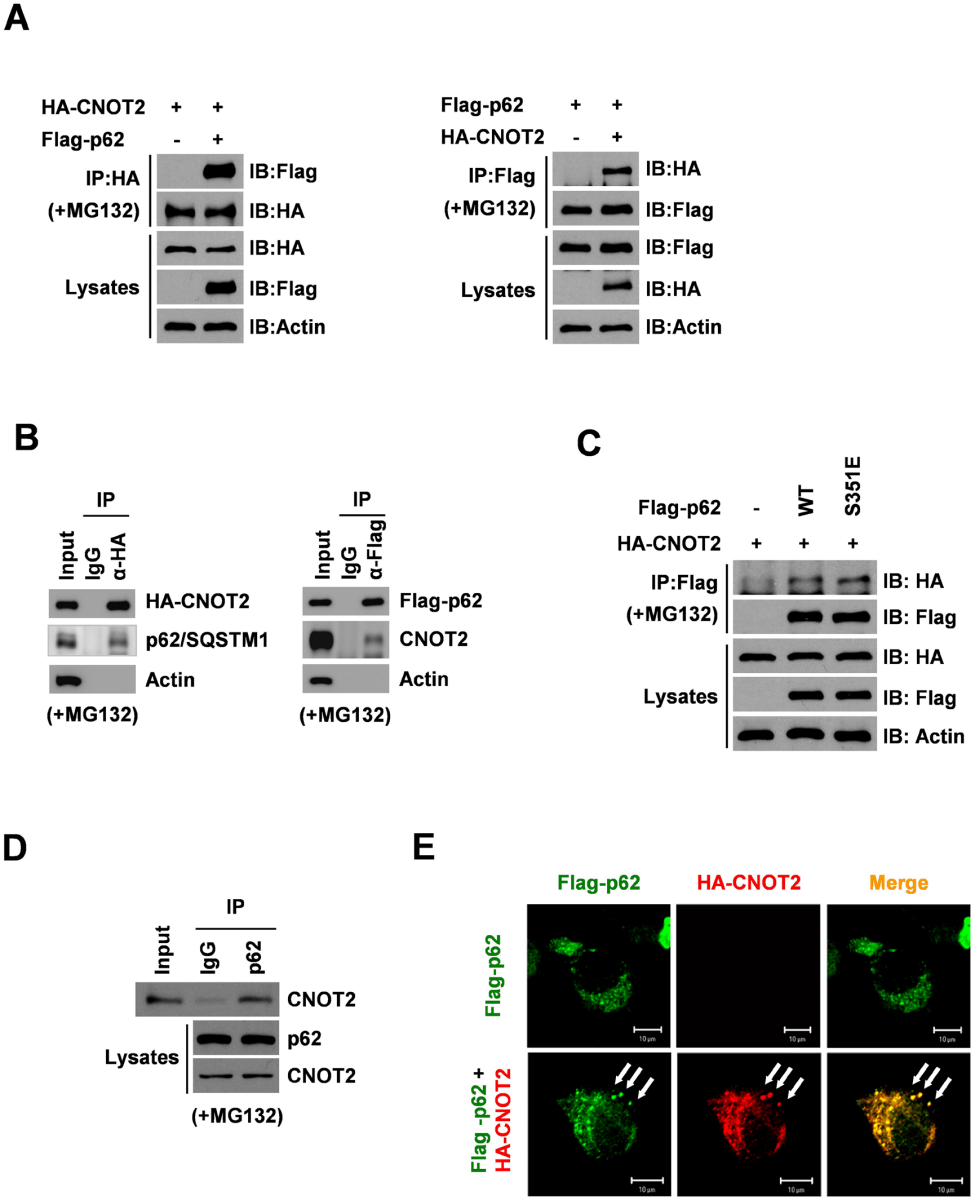
CNOT2 correlates with p62/SQSTM1 in HEK293 QBI cells by co-immunoprecipitation and immunofluorescence **(A)** HEK293 QBI cells were transiently transfected with Flag-p62 (4 μM) and/or HA-CNOT2 (4 μM). The transfected cells were treated with MG132 (10 μM) for 4 h before harvest and cell lysates were immunoprecipitated with HA-CNOT2 or Flag-p62 antibody and subjected to Western blotting. **(B)** HEK293 QBI cells were transiently transfected with HA-CNOT2 (4 μM) or Flag-p62 and exposed to MG132 (10 μM) for 4 h before harvest. Then cell lysates were immunoprecipiated with HA or Flag antibody and immunoblotted with antibodies of HA, Flag, p62, CNOT2 and actin. **(C)** HEK293 QBI cells were transiently transfected with HA-CNOT2 and/or Flag-p62 WT and Flag-p62 mutant S351 plasmids and treated with 10 μM MG132 for 4 h before cell harvest. Then cell lysates were immunoprecipitated with Flag-p62 antibody and subjected to Western blotting with antibodies of Flag and HA or Actin. **(D)** Immunoprecipitation of endogeneous CNOT2 with p62/SQSTM1 antibody. H1299 cells were treated 10 μM of MG132 for 4 h, cell lysates were precleaned with protein G/A beads and subsequently incubated for 1-2 h with protein G/A beads covalently coupled with anti-IgG and anti-p62/SQSTM1 antibody. **(E)** CNOT2 was colocalized with p62/SQSTM1. H1299 cells were transiently transfected with Flag-p62 (4 μg) and HA-CNOT2 (4 μg) plasmids (lower panel), followed by immunocytochemistry. The cells were stained with anti-p62 (green) and anti-CNOT2 (red). Bars, 10 μm.

To explore whether or not CNOT2 is a novel binding partner of p62/SQSTM1, we investigated the possibility that CNOT2 might be able to bind to p62/SQSTM1 by co-immunoprecipitation in HEK293 QBI cells. Here the correlation between CNOT2 and p62/SQSTM1 was detected in the presence of MG132 (Figure 3A and 3B). Next, the role of the phosphorylation of p62/SQSTM1 at serine 351(S351) was examined on correlation between CNOT2 and p62/SQSTM1 in HEK293 QBI cells. The S351 of p62-KIR, a phosphorylation-mimetic mutant is substituted with Glu (S351E), has a higher affinity for endogenous Keap1 than either wild-type p62 or the phosphorylation-defective S351A mutant [34]. As shown in Figure 3C, intriguingly, any significant difference was not detected between wild-type of p62/SQSTM1 and phosphorylation-mimetic S351E mutant for the interaction of CNOT2 and p62/SQSTM1, implying that the p62/SQSTM1 phosphorylation is not a prerequisite for CNOT2 and p62/SQSTM1 complex formation, regardless of Keap1 signaling. To rule out overexpression artifacts, HA-CNOT2 was immunoprecipitated with p62/SQSTM1 from H1299 whole-cell extracts. As a result, the interaction between endogenous CNOT2 and p62/SQSTM1 complex was still detected (Figure 3D). Consistently, confocal microscopy showed that CNOT2 was colocalized with p62/SQSTM1 in H1299 cells as shown in Figure 3E.

### The p62/SQSTM1 degradation by CNOT2 is mediated by ATG5

**Figure 4:**
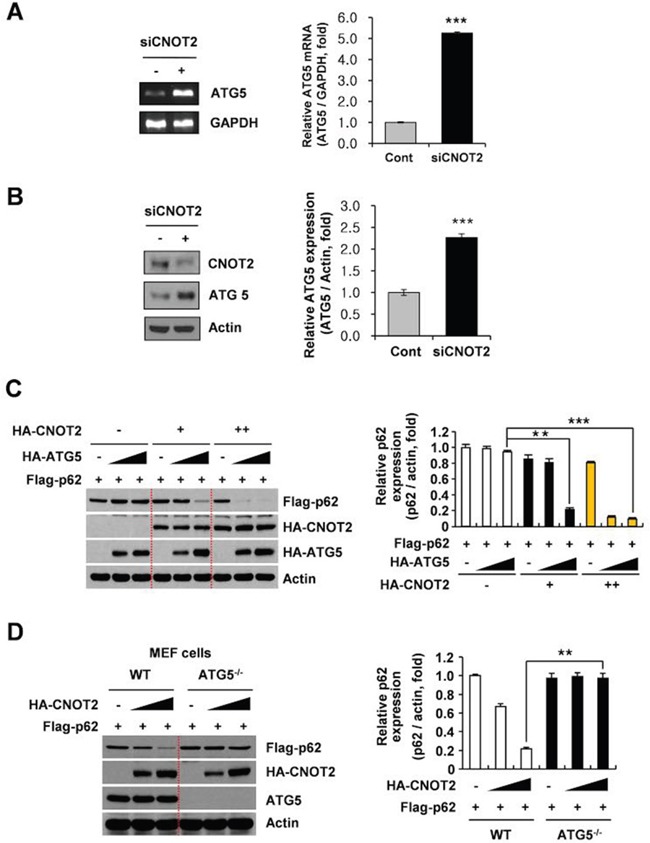
ATG5 is critically involved in CNOT2 induced degradation of p62/SQSTM1 in H1299 cells Effect of CNOT2 depletion on ATG5 was examined in H1299 cells transfected by CNOT2 siRNA at mRNA **(A)** and protein levels **(B)** by RT-PCR and Western blotting. Quantitative analysis for mRNA and protein levels for ATG5 in CNOT2-depleted H1299 cells (means ± SD of 3 independent experiments. **(C)** Effect of HA-CNOT2 and/or HA-ATG5 on p62 degradation in MEF cells transfected with Flag-p62 plasmid. MEF cells were transfected with HA-CNOT2 and/or HA-ATG5 and/or Flag-p62 plasmids for 24 h and then Western blotting was performed. **(D)** The crucial role of ATG5 in p62/SQSTM1 degradation by CNOT2 depletion in ATG5 wild and mutant MEF cells co-transfected with HA-CNOT2 and Flag-p62 plasmids by Western blotting.

Autophagosome formation is critically mediated by a set of ATG proteins [31, 35]. Thus, the important role of ATG5 was examined in CNOT2 induced degradation of p62/SQSTM1 in H1299 cells. RT-PCR and Western blotting revealed that the expression of ATG5 was increased in CNOT2-depleted H1299 cells at mRNA and protein levels (Figure 4A and 4B). To confirm the pivotal role of CNOT2 in the autophagic disposal of p62/SQSTM1, mouse embryonic fibroblast (MEF) cells were co-transfected with HA-CNOT2, HA-ATG5 and Flag-p62 expression constructs. Interestingly, ectopic expression of CNOT2 and ATG5 increased p62/SQSTM1degradation in a dose-dependent manner (Figure 4C). To confirm the crucial role of ATG5 in the degradation of p62/SQSTM1 by CNOT2, HA-CNOT2 and HA-p62 were co-transfected in ATG5 knock out (KO) MEF cells. Downregulation of p62 by CNOT2 was detected in MEF WT cells as expected, but not in ATG5 KO MEF cells (Figure 4D).

### PB1 domain of p62/SQSTM1 plays a crucial role in CNOT2 mediated p62/SQSTM1 degradation in H1299 cells

**Figure 5:**
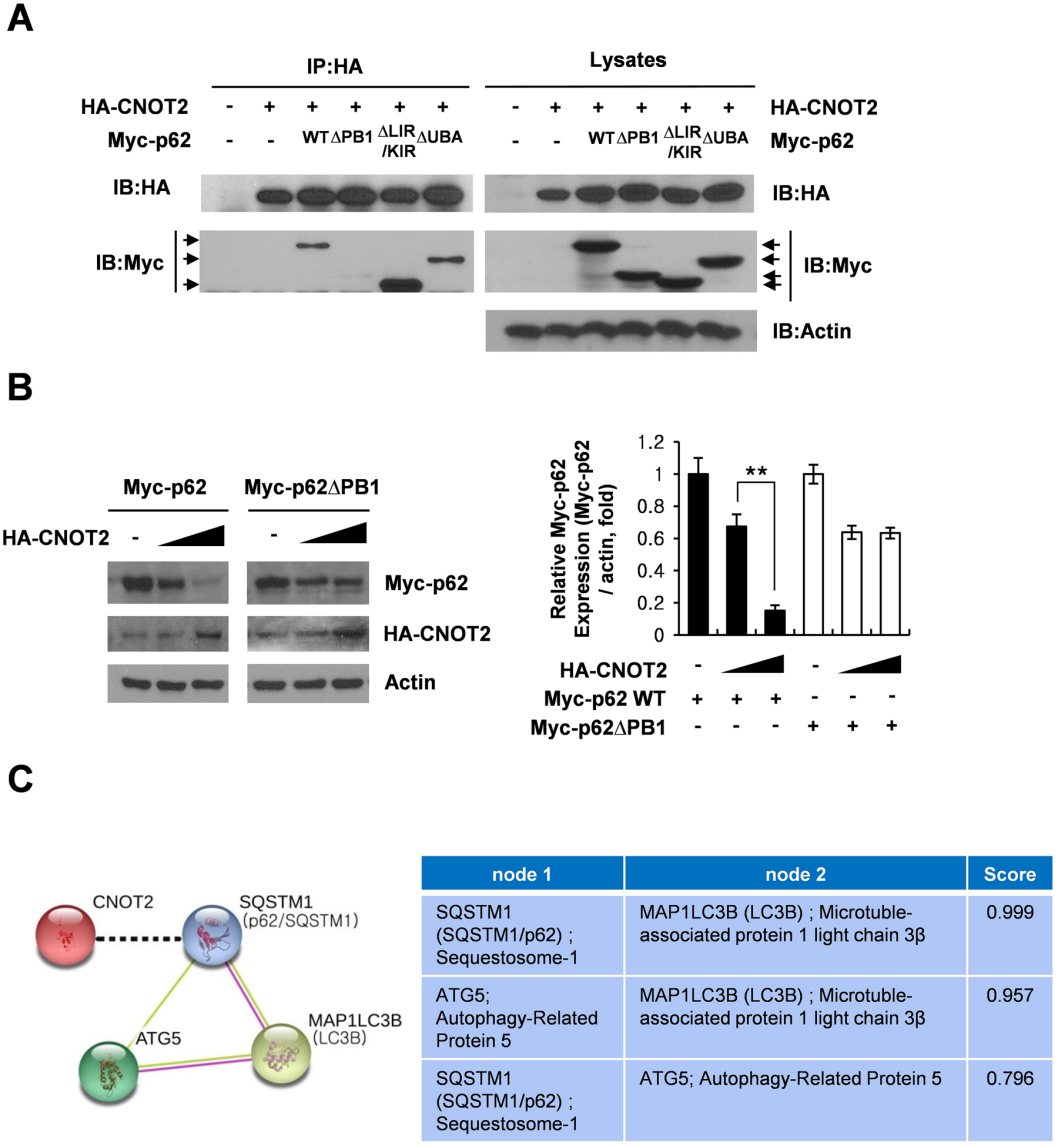
The pivotal role of p62 PB1 domain in CNOT2 induced degradation of p62/SQSTM1 and the effect of autophagy inhibitors on sub G1 population **(A)** HEK293 QBI cells were co-transfected with HA-CNOT2 and/or wild-type p62 (Myc-p62^wt^), p62 PB1 domain mutant (Myc-p62^ΔPB1^), p62 LIR/KIR domain mutant (Myc-p62^ΔLIR/KIR^) and p62 UBA domain mutant (Myc-p62^ΔUBA^) plasmids. Then the cell lysates were immunoprecipitated with HA antibody and subjected to Western blotting with Myc and HA antibodies. **(B)** The crucial role of p62 PB1 domain in HA-CNOT2 induced p62/SQSTM1 degradation in H1299 cells. H1299 cells were co-transfected with HA-CNOT2 and Myc-p62^wt^ or Myc-p62^ΔPB1^ plasmid and the cell lysates were subjected to Western blotting. **(C)** The prediction scores for PPI between p62/SQSTM1, ATG5 and LC3B regulated by CNOT2. The scores were represented by STRING using KEGG datasets, containing experimentally verified and putative PPIs. –, known interactions experimentally determined; –, Text Mining; --, suggested interaction in this study [37].

**Figure 6:**
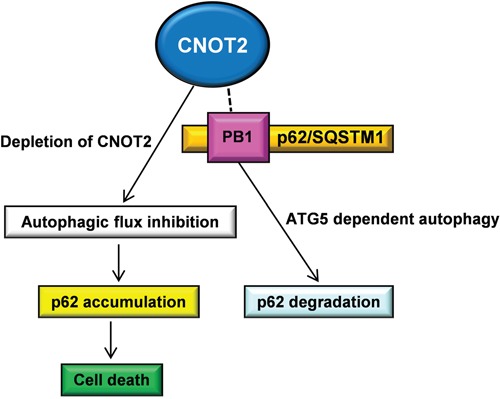
Schematic illustration for the correlation between CNOT2 and p62/SQSTM1 through ATG5 dependent autophagy

In general, p62 consists of several domains, such as an N-terminal PB1 domain, a ZZ-type zinc finger domain, a nuclear localization signal (NLS), an export motif (NES), the LC3-interacting region (LIR), the KEAP1-interacting region (KIR), and a C-terminal ubiquitin-associated domain (UBA), requisite for autophagic machinery and its related signaling pathways [5, 7, 36]. To investigate a pivotal role of specific p62 domain in CNOT2 induced degradation of p62/SQSTM1, HEK293 QBI cells were transfected with Myc-p62/SQSTM1 WT, Myc-p62/SQSTM1 ΔPB1, Myc-p62/SQSTM1 ΔLIR/KIR or Myc-p62/SQSTM1 ΔUBA together with a HA-CNOT2 and then subjected to co-immunoprecipitation. Here we found that HA-CNOT2 was efficiently co-immunoprecipitated with Myc-p62/SQSTM1 WT, Myc-p62/SQSTM1 ΔLIR/KIR or Myc-p62/SQSTM1 ΔUBA, but not with Myc-p62/SQSTM1 ΔPB1 (Figure 5A). To confirm the critical role of PB1 in CNOT2 induced degradation of p62, another Western blotting was performed in H1299 cells co-transfected with HA-CNOT2 and Myc-p62^wt^ or Myc-p62^ΔPB1^ construct. As shown in Figure 5B, co-transfection of Myc-p62^ΔPB1^ and HA-CNOT2 weakly induced p62 degradation in H1299 cells compared to Myc-p62^wt^ and HA-CNOT2 control. Also, as shown in Figure 5C the STRING database also supported our findings through the protein-protein interactions of p62/SQSTM1-LC3B, ATG5-LC3B and p62/SQSTM1-ATG5 with high binding scores of 0.999, 0.957 and 0.796, respectively [37]. Taken together, these findings suggest that CNOT2 induces degradation of p62/SQSTM1 mainly via interaction with p62/SQSTM1 ΔPB1 through ATG5 dependent autophagy (Figure 6), while CNOT2 depletion induces accumulation of p62/SQSTM1 leading to apoptotic cell death.

## DISCUSSION

In the current study, the critical role of CNOT2 was elucidated during autophagy. Here, CNOT2 depletion induced autophagic features of LC3B-II conversion, p62/SQSTM1 accumulation and increased number of puncta, implying the potential of autophagy induction. Nevertheless, autophagic flux was inhibited in CNOT2-depleted H1299 cells cotransfected with GFP-mRFP-LC3 construct in CNOT2-depleted H1299 cells. Consistently, TEM observation revealed some vacuoles with more autophagosomes and less autophagolysosomes, indicating the possibility of authphagic cell death via impaired autophagy in CNOT-depleted H1299 cells.

On the contrary, CNOT2 overexpression induced ubiquitination of p62/SQSTM1 in HEK293 QBI cells, demonstrating that CNOT2 induces degradation of p62/SQSTM1. Furthermore, the regulation of p62/SQSTM1 by CNOT2-mediated ubiquitination was blocked by an autophagy inhibitor 3-MA, but not by proteosome system, given that proteins are generally degraded mainly via the proteasome system for short lived proteins and macroautophagy for long-lived proteins [38].

Of note, p62 was clearly immunoprecipitated by CNOT2 in HEK293 QBI cells transfected with HA-CNOT2 and Flag-p62, implying the direct interactions between CNOT2 and p62/SQSTM1. Also, downregulation of p62/SQSTM1 by CNOT2 was not significantly enhanced by CHX treatment, implying protein synthesis is not critically involved in downregulation of p62/SQSTM1 by CNOT2.

Interestingly, CNOT2 was still detected by Western blotting of S351E mutant p62/SQSTM1 immunoprecipitates almost similar to wild-type of p62/SQSTM1 in HEK293 QBI cells transfected with S351E mutant p62/SQSTM1 plasmid, which has higher affinity to endogenous Keap1 rather than wild-type p62/SQSTM1 or the phosphorylation-defective S351A mutant p62/SQSTM1, implying that phosphorylation of p62/SQSTM1 is not critically involved in CNOT2 and p62/SQSTM1 complex formation regardless of Keap1 mediated pathway.

Notably, the expression of ATG5 was upregulated at mRNA and protein levels in CNOT2-depleted H1299 cells. Furthermore, ectopic expression of CNOT2 and ATG5 enhanced p62/SQSTM1 degradation in a dose-dependent manner, demonstrating that p62/SQSTM1 degradation by CNOT2 is mediated by ATG5 for autophagosome formation. Consistently, degradation of p62/SQSTM1 by CNOT2 was detected in *ATG5^+/+^* MEF cells, but not in *ATG5^−/−^* MEF cells, implying the potential role of ATG5 in CNOT2 induced degradation of p62/SQSTM1.

Interestingly, co-immunoprecipitation revealed that CNOT2 effectively binds to Myc-p62/SQSTM1 WT or Myc-p62/SQSTM1 ΔLIR/KIR or Myc-p62/SQSTM1 ΔUBA, but not to Myc-p62/SQSTM1 ΔPB1 in HEK293 QBI cells transfected with Myc-p62/SQSTM1 WT, Myc-p62/SQSTM1 ΔPB1, Myc-p62/SQSTM1 ΔLIR/KIR and Myc-p62/SQSTM1 ΔUBA with HA-CNOT2. Consistently, Western blotting showed that cotransfection of Myc-p62^ΔPB1^ and HA-CNOT2 weakly induced p62 degradation in H1299 cells compared to Myc-p62^wt^ and HA-CNOT2 control, indicating that p62 PB1 domain is prerequisite in CNOT2 induced degradation of p62/SQSTM1. However, considering previous evidences that the autophagy lysosomal pathway is dependent on the polymerization of p62/SQSTM1 via the NH2-terminal PB1 domain and polyubiquitin binding via the COOH-terminal UBA domain of p62/SQSTM1 [39], the role of UBA domain should be further examined in CNOT2 induced degradation of p62/SQSTM1 in the future.

It is well documented that several apoptotic proteins such as PUMA, Bax, XIAP, and Bim regulate autophagy and also autophagic proteins for nucleation and elongation modulate apoptosis through caspase and calpain-mediated cleavage of ATGs, implying crosstalk between apoptosis and autophagy. Likewise, accumulation of p62/SQSTM1 is critically involved in determination of the fate of cell through the caspase dependent apoptotic pathway [2, 9, 16]. Herein, sub G_1_ population was efficiently increased in CNOT2-depleted H1299 cells by late stage autophagy inhibitors such as NH_4_Cl and CQ compared to an early stage autophagy inhibitor 3-MA in CNOT2-depleted H1299 cells, indicating depletion of CNOT2 induces apoptotic cell death via impaired autophagy with increased autophagosomes.

Collectively, CNOT2 depletion increased p62/SQSTM1 accumulation, LC3B-II conversion and the number of puncta, but impaired autophagic flux in H1299 cells, along with TEM observation of more autophagosomes and less autophagolysosomes. ATG5 played a pivotal role in CNOT2 induced degradation of p62/SQSTM1 only in *ATG5^+/+^* MEF cells, but not in *ATG5^−/−^* MEF cells, while PB1 domain of p62 is essential in CNOT2 induced degradation of p62/SQSTM1. Furthermore, apoptotic cell death was more efficiently exhibited in CNOT2-depleted H1299 cells by NH_4_Cl and CQ compared to 3-MA in CNOT2-depleted H1299 cells. Overall, these findings provide novel insight into the critical role of CNOT2 as a negative autophagy regulator in ATG5 dependent pathway.

## MATERIALS AND METHODS

### Reagents and antibodies

Dulbecco's modified Eagle's medium (DMEM, LM001-05), fetal bovine serum (FBS, S001-07) and Roswell Park Memorial Institute (RPMI, LM011-01) 1640 medium were purchased from Welgene (GD, Korea). 3-methyladenine (3-MA, 5142-23-4), Z-Leu-Leu-Leu-al (MG132, C221) and cycloheximide (CHX, 01810) were purchased from Sigma-Aldrich (St Louis, MO, USA). X-tremeGENE HP DNA transfection reagents (06366236001) were purchased from Roche (Mannheim, Germany). DC Protein Assay Kit II (500-0113, 0114 and 0115) was purchased from Bio-Rad (Hercules, CA, USA). ECL western blotting detection reagents (RPN2209) were purchased from GE Healthcare (Buckinghamshire, UK). Antibodies for CNOT2 (34214, 1:2000 dilution), p62/SQSTM1 (5114, 1:2000 dilution), Myc-taq (2276, 1:2000 dilution), LC3B (3868, 1:1000 dilution) and ATG5 (8540, 1:2000 dilution) were purchased from Cell Signaling Technology (Beverly, MA, USA). Antibodies for OctA-taq (Flag, SC-807, 1:2000 dilution), HA-taq (SC-7392, 1:2000 dilution), immunoglobulin G (IgG, SC-69786), actin (SC-47778, 1:5000 dilution), 4′, 6′-diamidino-2′-phenylindole dilactate (DAPI, 28718-90-3) and Protein A/G plus-agarose (SC-2003) were purchased from Santa Cruz Biotechnology (Dallas, Texas, USA). Other chemical reagents were obtained from Sigma-Aldrich.

### Plasmids

The cDNA encoding C-terminal 440 amino acids of wild-type and S351E (a phosphorylation mimic) p62/SQSTM1 were subcloned into the pcDNA vector (Invitrogen, Waltham, MA, USA) for protein expression, which tagged the protein with an Flag-tag at the C-terminal end. HA-CNOT2 was kindly provided by Dr. KS Kim (Yonsei University, Seoul, Korea). HA-ubiquitin expression vector was kindly supplied by Dr. J Ha, (Kyung Hee University, Seoul, Korea). GFP-LC3 and RFP-LC3 were kindly provided by Dr. H Hu (China Agricultural University, Beijing, China). The plasmids of pcDNA3.1-Myc-p62, ΔPB1, ΔLIR/KIR and ΔUBA were kindly supplied by prof. SH Bae (Yonsei University, Seoul, Korea).

### Cell culture and DNA transfection

H1299 (ATCC^®^ CRL-5803D™; human non-small cell lung carcinoma) cells were obtained from American Type Culture Collection (ATCC) and maintained in RPMI 1640 supplemented with 10 % FBS. *ATG5 ^+/+^* and *ATG5^−/−^* MEF and HEK293 QBI (human embryonic kidney 293; ATCC^®^ CRL-1573™) cells provided by Prof. J Ha (Kyung Hee University, Seoul, Korea) were maintained in DMEM supplemented with 10 % FBS. HEK293 QBI cell line is a superior subclone of HEK293 QBI cells, harboring the E1A and E1B regions of the adenoviral genome, and complementing the deletion of the E1 region in the recombinant Adenovirus (Qbiogene, Livingstone, UK). All cells were maintained in the growing medium in a humidified 5 % CO_2_ atmosphere at 37°C. DNA transfection was performed using the X-tremeGENE HP DNA transfection reagents (Roche, Quebec, Canada).

### Small interfering RNA (siRNA) transfection

H1299 cells were transfected with siRNA oligoribonucleotides targeted against human CNOT2 or a RNA interference negative control. Each well was incubated for 48 h with 1 nM of siRNA using INTERFERIN siRNA transfection reagent (409-50, Polyplustransfection Inc., NY, USA) according to the manufacturer's protocol. The cells were then washed off from the plates and transferred into serum-free medium for treatments.

### Western blotting and co-immunoprecipitation (co-IP)

H1299 cells or HEK293 QBI cells were washed twice with cold PBS and harvested by scraping with a rubber policeman. The cells were pelleted by centrifugation at 4°C and resuspended directly into a lysis buffer (50 mM Tris-HCl, pH 7.4, 150 mM NaCl, 1 % Triton X-100, 2 mM EDTA, 0.5 % IGEPAL CA-630, 10 mM NaF, 2 mM Na_3_VO_4_, and 0.01 % protease inhibitor cocktail). Cell lysates were subjected to SDS-PAGE and transferred to a nitrocellulose membrane. After blocking in 5 % skim milk and Tris-buffered saline with 0.1 % Tween-20 (TBST 10X), signals were detected and analyzed by a Kodak X-OMAT 2000 image analyzer. For immunoprecipitation experiment, cell lysates were precleared with protein A/G-agarose beads and subsequently incubated for an 1 - 2 h with protein G/A beads covalently coupled with anti-HA, anti-Flag and anti-Myc antibodies. Immune complexes were washed four times with cell extraction buffer. Eluted samples or whole cellular lysates were resolved by SDS-PAGE and proteins were detected by Western blotting using the indicated antibodies. Then densitometric analysis was performed using ImageJ software.

### Immunocytochemistry

For immunostaining, H1299 cells were grown on coverslips to 70 % confluency. Cells were fixed in 3.7 % formaldehyde in PBS for 15 min at room temperature and permeabilized with 0.2 % Triton X-100 (9002-93-1, Amresco, OH, USA) in phosphate-buffered saline (PBS) for 10 min and blocked with 1 % BSA (82-100-6, Millipore, MA, USA) in PBS for 1 h. The fixed cells were incubated for 2 h with anti-LC3 primary antibodies, and then washed in PBS and incubated with Alexa Fluor^®^ 488-conjugated anti-rabbit IgG antibody (A-11034, Invitrogen, CA, USA) for 1 h. The cells were then stained with 0.5 mg/ml of DAPI to visualize nuclei. Cells were washed in PBS, mounted on glass slides and observed with a LSM510 confocal laser microscope (Carl Zeiss, Heidelberg, Germany).

### Transmission electron microscopy (TEM) analysis

H1299 cells were fixed in 2 % paraformaldehyde (158127, Sigma) and 1 % (Osmium tetroxide (O_s_O_4_) solution in 0.05 M sodium cacodylate buffer (pH 7.2) at 4°C. Samples were stained in 0.5 % uranyl acetate for 30 min and dehydrated in increasing concentrations of ethanol. Then, cells were infiltrated with Spurr's resin and dried in an oven at 70°C for 24 h. The samples were sectioned with ultramicrotome (Tucson, USA) and stained with 2 % uranyl acetated and Reynolds’ lead citrate. The samples were then observed with a TEM (Carl Zeiss, Germany).

### Autophagic flux assay

For autophagic flux assay, GFP-mRFP-LC3 construct was stably transfected into H1299 cells using the X-treme GENE HP DNA transfection reagent. The cells were washed twice in ice-cold PBS, fixed, mounted with Histological Mounting Medium (HistoMount™, HS-103, National diagnostics, Atlanta, USA) and observed with LSM510 confocal laser microscope (Carl Zeiss,, Heidelberg, Germany).

### Ubiquitination assay

HEK293 QBI cells were transiently co-transfected with HA-CNOT2, Flag-p62 and Myc-ubiquitin plasmids in the presence or absence of 5 mM 3-MA for 24 h and 10 μM MG132 for 4 h before harvest. For analysis of ubiquitination of endogenous p62/SQSTM1, the cells were lysed with lysis buffer and immunoprecipitated with anti-Myc antibody. The ubiquitination reaction mixtures were collected by centrifugation and washed three times with lysis buffer. The samples were then subjected to SDS-PAGE followed by Western blotting. Ubiquitination of p62/SQSTM1 was analyzed by Western blotting with anti-p62/SQSTM1 antibody.

### Reverse transcription polymerase chain reaction (RT-PCR)

Total cellular RNA was extracted from the cells using the QIAzol lysis reagent (79306, QUAZEN, Germantown, USA). A cDNA was synthesized from 2 mg of total RNA using M-MLV reverse transcriptase (RT001S, Enzynomics, DJ, Korea). Specific primers for RT-PCR: p62/SQSTM1 (sense) 5′- TCAACTTC AATGCCCAGAGG-3′ and (antisense) 5′- CTGCCC AGACTACGACT TGTGT-3′; ATG5 (sense) 5′- AGCAACTCTGA GTGGGATTG-3′ and (antisense) 5′- CAC TGCAGAGGTGTTTCCAA - 3′; GAPDH (sense) 5′- TCACCATCTTCC AGGAGCGA - 3′ and (antisense) 5′- CACAATGCCGAAGTG GTCGT - 3′. PCR products were analyzed by agarose gel electrophoresis and visualized using ethidium bromide. The detected bands were quantitated using Image J software (National Institutes of Health, Bethesda, MD, USA).

### Flow cytometric analysis

For cell cycle analysis, H1299 cells were transfected with CNOT2 siRNA for 48 h and then treated with 3-MA, chloroquine (CQ, Sigma, C6628) and ammonium chloride (NH_4_Cl, Sigma, 12125-02-9) for another 24 h. The cells were fixed with 75 % ethanol at -20 ^°^C, washed twice with PBS and resuspended in PBS containing RNase A (1 mg/ml, Sigma, R6513) and incubated for 1 h at 37 ^°^C. After incubation, the cells were stained with 500 μl of propidium iodide (50 mg/ml, Sigma, P4170) for 30 min at room temperature in the dark. The DNA contents of the stained cells were analyzed using CellQuest Software with the FACSCalibur flow cytometer (Becton Dickinson, Franklin Lakes, NJ, USA).

### Statistical analysis

The results were expressed as the means ± SD from at least three independent experiments. Statistical analyses were conducted by Student's *t*-test using SigmaPlot version 12 (Systat Software Inc., San Jose, CA, USA). P value < 0.05 was considered statistically significant.

## Abbreviations

CCR4-Not complex: carbon catabolite repressed 4-negative on TATA-less complex
p62/SQSTM1: p62 sequestosome 1
LC3: microtubule-associated protein 1A/1B-light chain 3
ATG5: autophagy-related gene 5
3-MA: 3-methyladenine
CHX: cycloheximide
MG132: carbobenzoxy-Leu-Leu-leucinal
DAPI: 4′, 6′-diamidino-2′-phenylindole dilactate
TEM: transmission electron microscopy
HRP: horseradish peroxidase
PARP: poly (ADP-ribose) polymerase
Caspase: cysteine aspartyl-specific protease
3-MA: 3-methyladenine
CQ: chloroquine
NH_4_Cl: ammonium chloride
MEF: mouse embryo fibroblast
PPI: protein-protein interactions
PB1: Phox and Bem1p
UBA: ubiquitin-associated domain
