# Correction: CNOT2 promotes degradation of p62/SQSTM1 as a negative regulator in ATG5 dependent autophagy

**DOI:** 10.18632/oncotarget.27211

**Published:** 2019-09-17

**Authors:** Kwon Jeong, Hee Young Kwon, Myoung Seok Jeong, Eun Jung Sohn, Sung-Hoon Kim

**Affiliations:** ^1^ Cancer Molecular Targeted Herbal Research Center, College of Korean Medicine, Kyung Hee University, 1 Hoegi-Dong, Dongdaemun-Gu, Seoul 130-701, Republic of Korea

**This article has been corrected:** Due to errors in image preparation, the IP blots in Fig. 3A contained identical images. These duplicates have now been replaced. The proper Figure 3A is shown below. The authors declare that these corrections do not change the results or conclusions of this paper.

Original article: Oncotarget. 2017; 8:46034–46046. 46034-46046. https://doi.org/10.18632/oncotarget.17682

**Figure 3:**
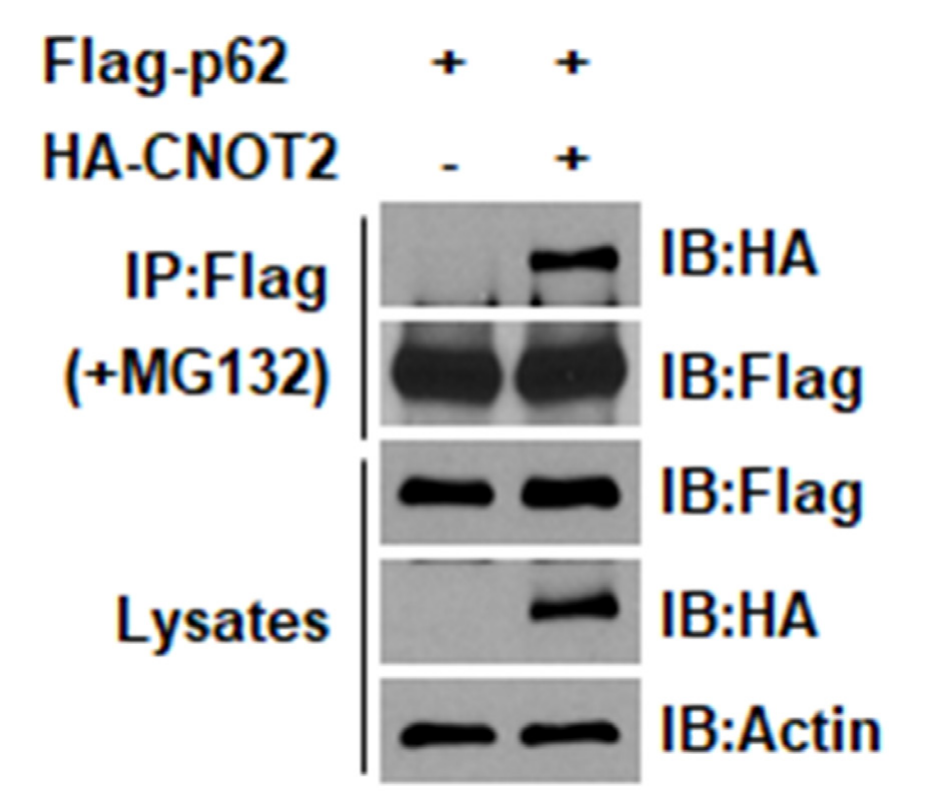
CNOT2 correlates with p62/SQSTM1 in HEK293 QBI cells by co-immunoprecipitation and immunofluorescence. (A) HEK293 QBI cells were transiently transfected with Flag-p62 (4 μM) and/or HA-CNOT2 (4 μM). The transfected cells were treated with MG132 (10 μM) for 4 h before harvest and cell lysates were immunoprecipitated with HA-CNOT2 or Flag-p62 antibody and subjected to Western blotting. (B) HEK293 QBI cells were transiently transfected with HA-CNOT2 (4 μM) or Flag-p62 and exposed to MG132 (10 μM) for 4 h before harvest. Then cell lysates were immunoprecipiated with HA or Flag antibody and immunoblotted with antibodies of HA, Flag, p62, CNOT2 and actin. (C) HEK293 QBI cells were transiently transfected with HA-CNOT2 and/or Flag-p62 WT and Flag-p62 mutant S351 plasmids and treated with 10 μM MG132 for 4 h before cell harvest. Then cell lysates were immunoprecipitated with Flag-p62 antibody and subjected to Western blotting with antibodies of Flag and HA or Actin. (D) Immunoprecipitation of endogeneous CNOT2 with p62/SQSTM1 antibody. H1299 cells were treated 10 μM of MG132 for 4 h, cell lysates were precleaned with protein G/A beads and subsequently incubated for 1-2 h with protein G/A beads covalently coupled with anti-IgG and anti-p62/SQSTM1 antibody. (E) CNOT2 was colocalized with p62/SQSTM1. H1299 cells were transiently transfected with Flag-p62 (4 μg) and HA-CNOT2 (4 μg) plasmids (lower panel), followed by immunocytochemistry. The cells were stained with anti-p62 (green) and anti-CNOT2 (red). Bars, 10 μm.

